# Giant scrotal swelling in association with a congenital giant melanocytic nevus: A case report

**DOI:** 10.1016/j.jpra.2020.10.003

**Published:** 2020-10-27

**Authors:** Yasser M. Elkiran, Mohammed A. Abdelmaksoud, Mohamed S. Abdelgawwad, Nshaat A. Elsaadany, Amr M. Elshafei

**Affiliations:** Department of vascular and Endovascular surgery, Mansoura University hospitals, Mansoura University, Egypt

**Keywords:** Scrotal, Lymphedema, Reconstruction, Scrotoplasty

## Abstract

**Introduction:**

Scrotal lymphedema is a rare condition, with significant psychological and functional disability. To date, association with giant congenital melanocytic nevus has not been reported.

**Case report:**

We report a case of a 15-year-old male with a giant congenital nevus associated with giant scrotal lymphedema. Surgical debulking with penoscrotoplasty achieved satisfactory functional and esthetic results.

**Conclusions:**

Early diagnosis and surgical intervention should be advocated for congenital causes of large scrotal swelling.

## Introduction

Lymphedema is a chronic disease characterized by progressive deposition of protein-rich fluid in the subcutaneous tissue due to a variety of causes leading to impairment of the lymphatic drainage system. The causes vary between congenital and acquired conditions. It may affect any part of the body.^1^ The scrotum can be affected by this condition and can be the cause of significant psychological and functional disability. A wide range of procedures have been described, but without huge success towards cure.^2^ Giant congenital melanocytic nevus (GCMN) is a rare skin lesion, with a reported incidence of one per 20,000. It is predominantly a condition of young females, with no reports in the literature to date involving male genitalia. GCMN is a variant of the common nevus that can be subdivided into junctional, intradermal, or compound variants.^3^ We present the case of giant scrotal lymphedema associated with GCMN which posed a treatment and reconstruction challenge.

## Case report

**Fig. 1:**
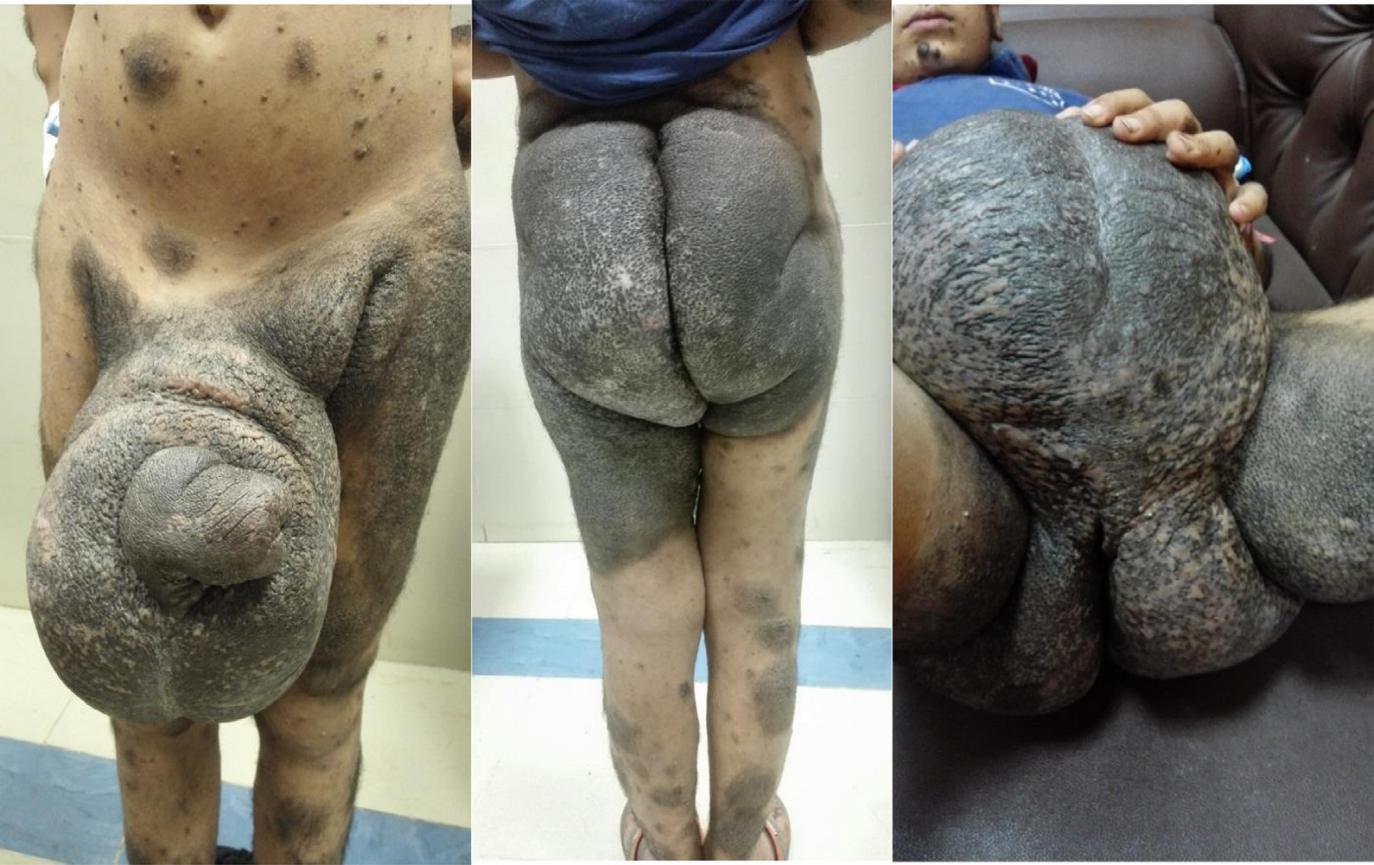
15-years-old child with giant scrotal swelling in association with giant melanocytic nevus, involving most of the back, perineum and inner thighs.

A 15-year-old non-circumcised male patient presented with a history of dark skin discoloration and associated thickening since birth, affecting various parts of body but mainly the scrotum, perineum, both groins and buttocks. The parents reported progressive scrotal swelling when he was three years old. Over the years, the patient suffered from recurrent skin infections and ulcerations affecting the scrotal skin. There were no problems related to micturition. No history of other diseases or operations were documented. On initial presentation, the enlarged scrotum approximated the size of a basketball (Fig. 1), impeding the proper fitting of clothing.

Physical examination revealed multiple black hyperpigmented lesions all over the body extensively involving the external genitalia; groin, upper thigh, perineum and buttocks (Fig 1). The circumference of scrotum was 73 cm. Right and left testicles could not be palpated.

The penile skin was lymphedematous and empty (Fig. 1). Immunochromatographic Test (ICT) results showed negative findings for filarial parasites. MRI and scrotal ultrasonography showed edema of the scrotum with a skin thickness of 5–6 cm. The penis could not be identified either by US or MRI. After extensive counseling with the patient and his family, a multi-disciplinary decision was made in favor of surgical reconstruction after control of any skin infections.

**Fig. 2:**
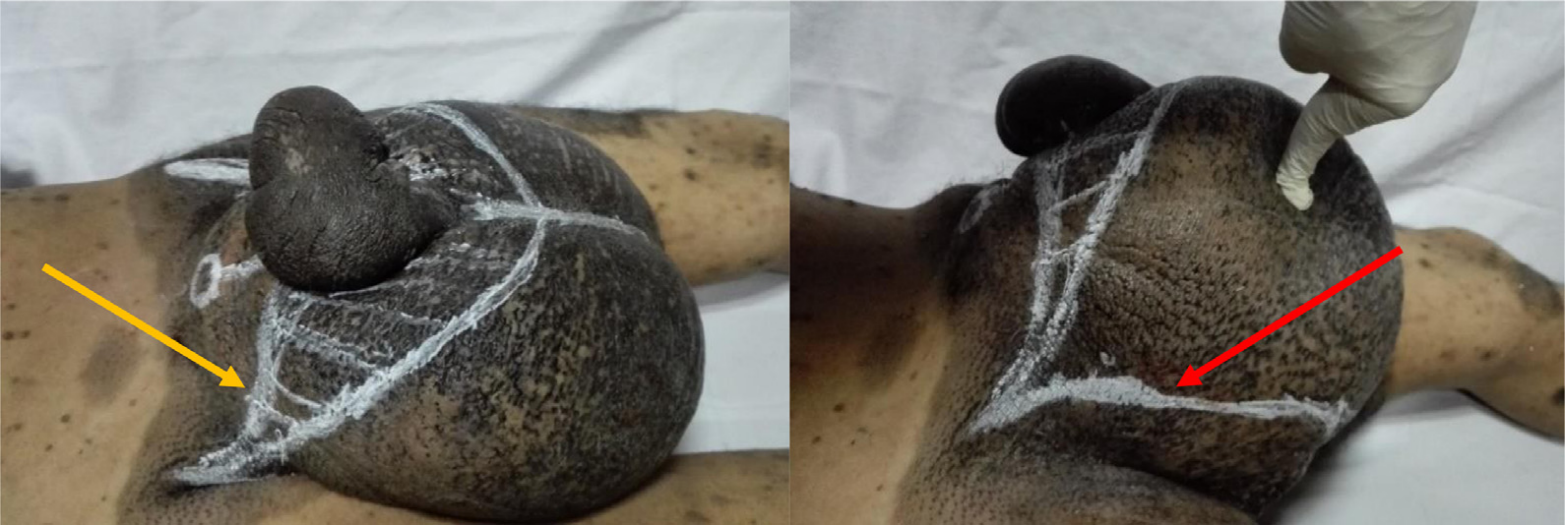
Our smile-like incision; its upper line (yellow arrow) started from the scrotal neck from one side and passes below the penoscrotal sulcus by 1 inches (shifted proximally after identification the site of the penis) and ended at the neck of the scrotum over the external inguinal ring on the opposite side, the lower line of the smile-like incision started from the same point and curved posteriorly and laterally (red arrow) reaching the other endpoint through unhealthy scrotal skin.

**Fig. 3:**
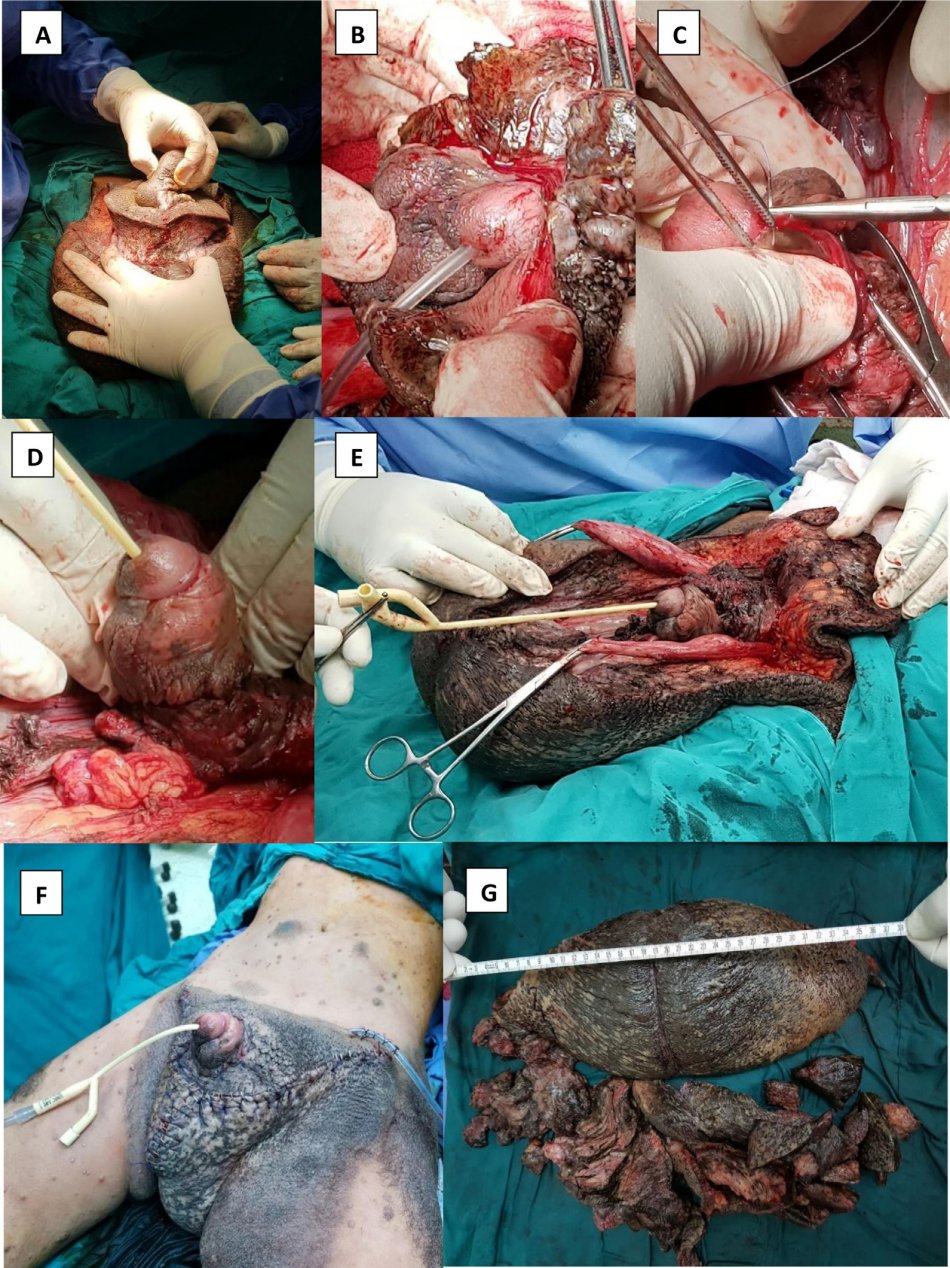
Operative images;Upper line of the smile-like incision and thick subcutaneous tissue(A), incision of the penile skin along its ventral raphe cautiously from distal to proximal with secured penile urethra by urinary Cather(B), eversion of the preputial skin after careful dissection of the penile shaft(C and D), eversion of the tunica vaginalis after careful dissection of the spermatic cord and the testis.(E)Final image after completed scrotoplasty(F) and removed unhealthy lymphedematous tissue (G).

Our previously reported smile-like incision^4^ was planned, with the upper incision line starting from the neck of the scrotum overlying the external inguinal ring from one side and passing an inch below the penoscrotal sulcus and ending at the neck of the scrotum over the external inguinal ring on the opposite side. The lower incision line of the smile-like incision started from the same point and curved posteriorly and laterally reaching the other endpoint through unhealthy scrotal skin. The upper line of the incision was again shifted proximally after identification of the site of the penis. (Fig. 2) We removed most of the unhealthy scrotal skin and subcutaneous tissues including the septum. Eversion of the tunica vaginalis was performed after careful dissection of the spermatic cord and testis. The penile shaft could not be identified through the hard-fibrotic subcutaneous tissue. In order to identify the penile shaft, we incised the penile skin along its ventral raphe from distal to proximal. The glans was identified protruding through the penoscrotal sulcus. The inner layer of the prepuce which was healthy was dissected, and was used to partially cover the penile shaft. The penis was brought through a button hole opening in the upper flap with final trimming of the skin flaps and closure of the scrotoplasty. A single suction drain was used. (Fig. 3)

Histopathology revealed a compound melanocytic nevus with deep tissue involvement, no cytological atypia and the cells were S-100 and HMB 45 positive.

## Discussion

Genital pigmented lesions arise mainly on the vulva (labia majora, labia minora, and clitoris), although they may occur less often on the perineum, pubic region, and male genitalia (penis and scrotum).^5^ Friedman and Ackerman^6^ first described an atypical melanocytic nevus of genital type in a series of seven unusual vulvar nevi. A previous study by Clark et al.^7^ described clinical and histological features of genital lesions and recommended local excision as the preferred treatment. Arao et al.^8^ reported a giant congenital nevus in a 32-year-old woman who had a massive pigmented tumor of the vulva. It grew over a period of eight years and was histologically composed of benign nevus-like cells with focal areas of extensive fibrous response.

In a review of the literature, no similar scrotal lesions were reported. This case describes the occurrence of a congenital melanocytic nevus as a large scrotal mass. Biopsy remains the mainstay of diagnosis in cases of scrotal lesions along with the aid of immunohistochemistry.

**Fig. 4:**
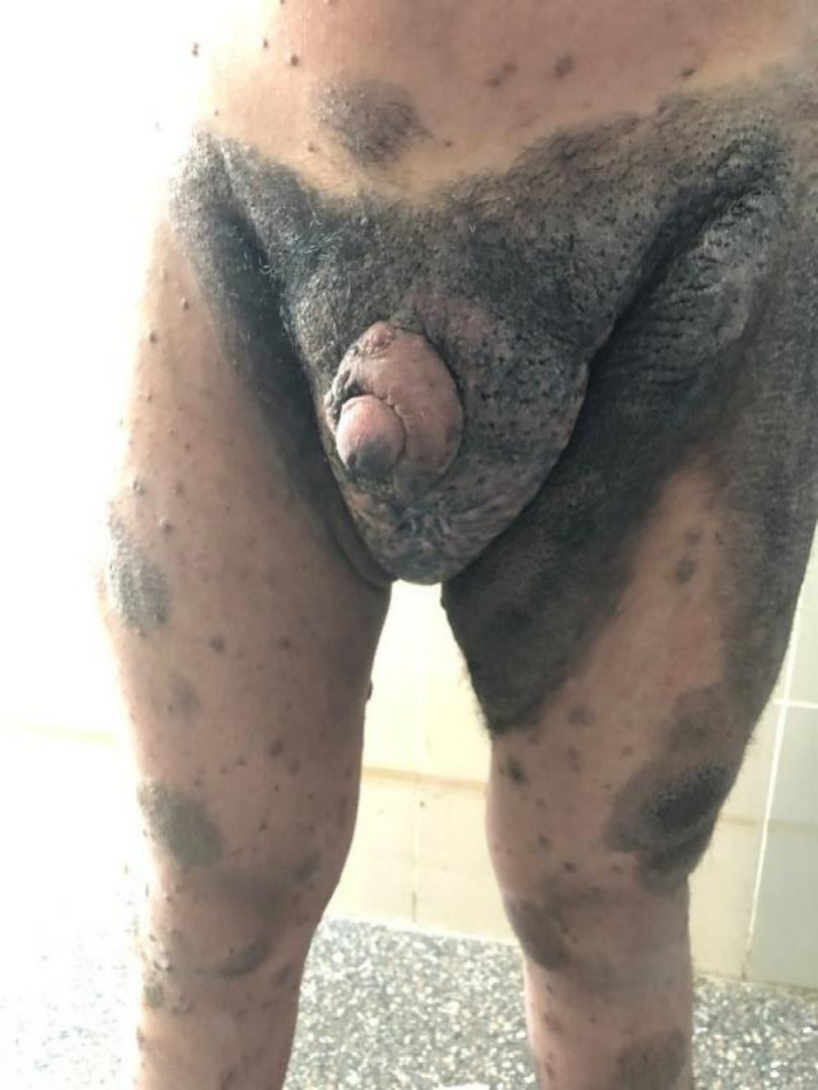
Postoperative follow up after one year.

Debulking surgery with local excision and reconstruction of the external genitalia is the preferred treatment. These lesions have a low risk of local recurrence and no further adverse outcome as they have a low malignant potential. A major challenge is to reconstruct the covering of the penile shaft, for which a large number of surgical techniques and procedures have been reported.^9^ The application of a split-thickness skin graft (STSG) over the penile shaft promotes adequate skin coverage with minimal attenuation of penile sensation. A STSG is compatible with sexual intercourse. This seems to produce better results than the use of flaps, even local flaps can affect tactile sensitivity and erection. However, patients whose shaft was covered with prepucial skin reported better penile sensitivity than those whose penile shaft was covered with a STSG.^10^ Patients, whose prepucial skin was sacrificed, had mild pain during erection that persisted for 1 year, after which this complaint gradually lessened. Surprisingly, this complaint was not encountered in patients whose inner prepucial skin was preserved.^11^ In our case, we preserved prepucial skin for coverage of most of the penile shaft. (Fig. 4)

## Conclusion

Giant penoscrotal lymphedema is a rare entity. Surgery is the most appropriate therapeutic option whatever the cause. Presentation of giant melanocytic nevus in association with giant scrotal swelling is very rare. Debulking of the diseased skin and subcutaneous tissue followed by penoscrotoplasty with coverage of distal penile shaft using prepucial skin gave an adequate and satisfactory cosmetic and functional outcome.

## Declaration of Competing Interest

None declared.
